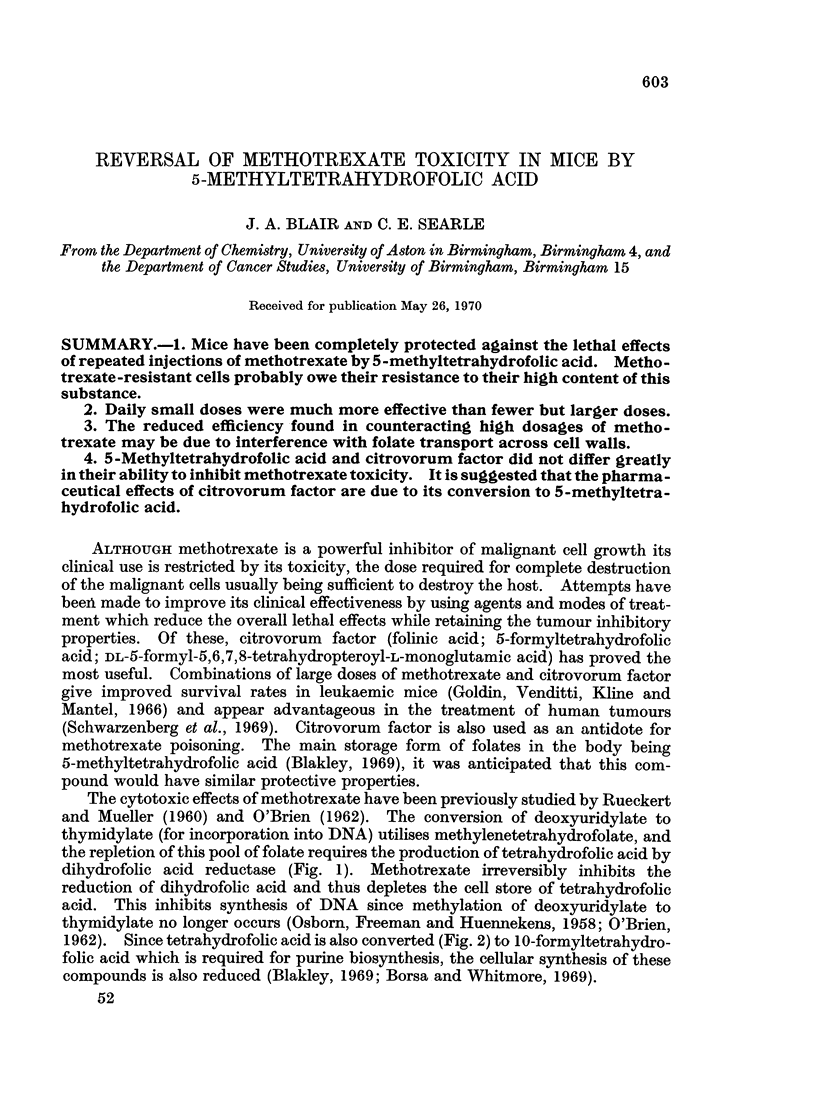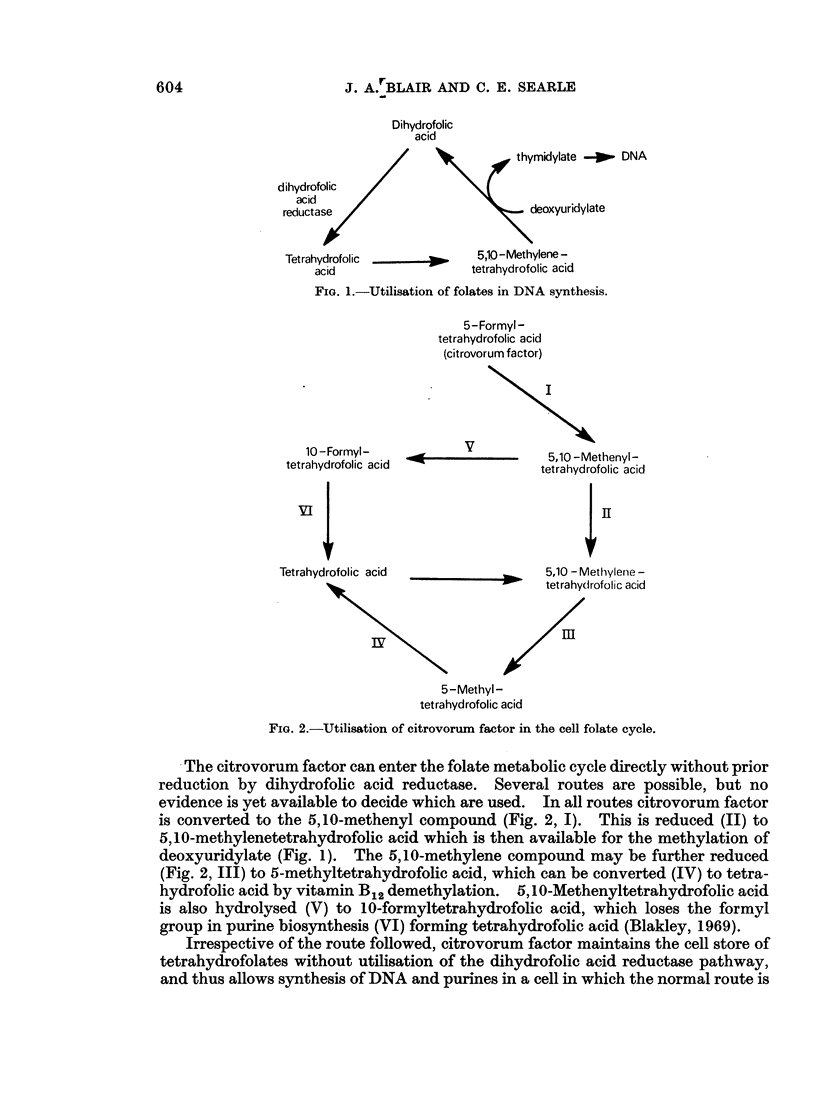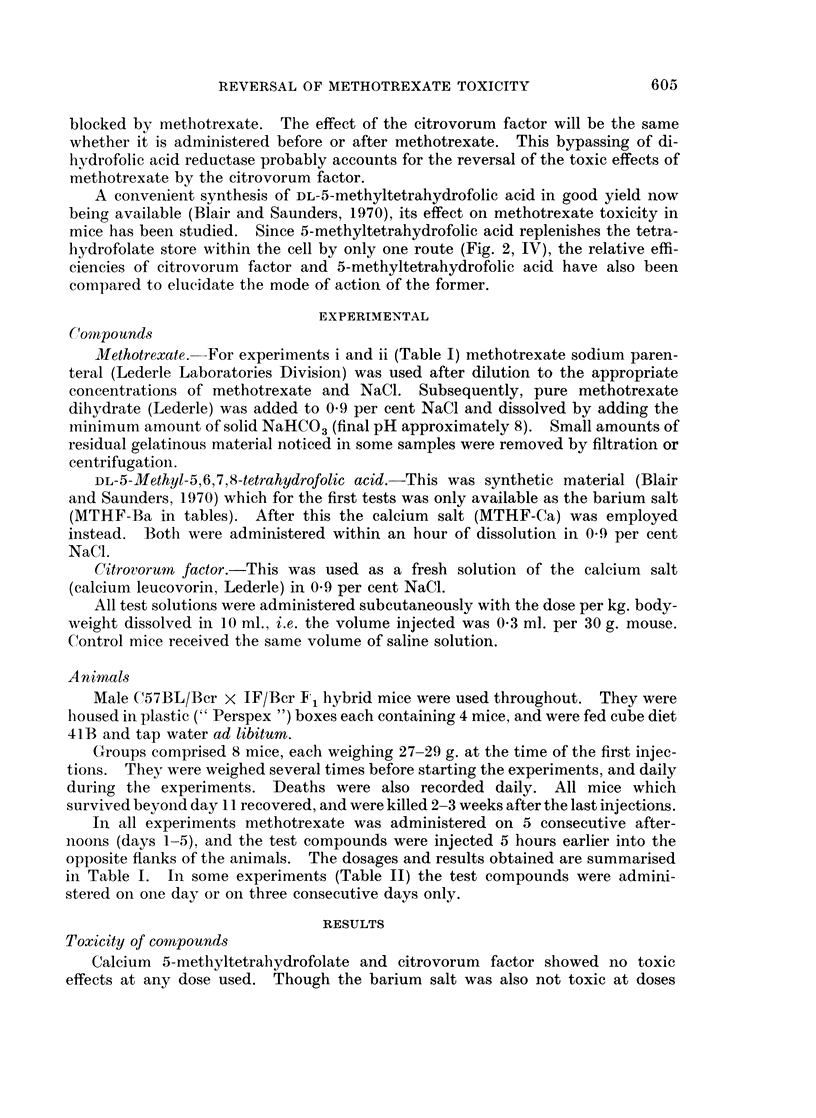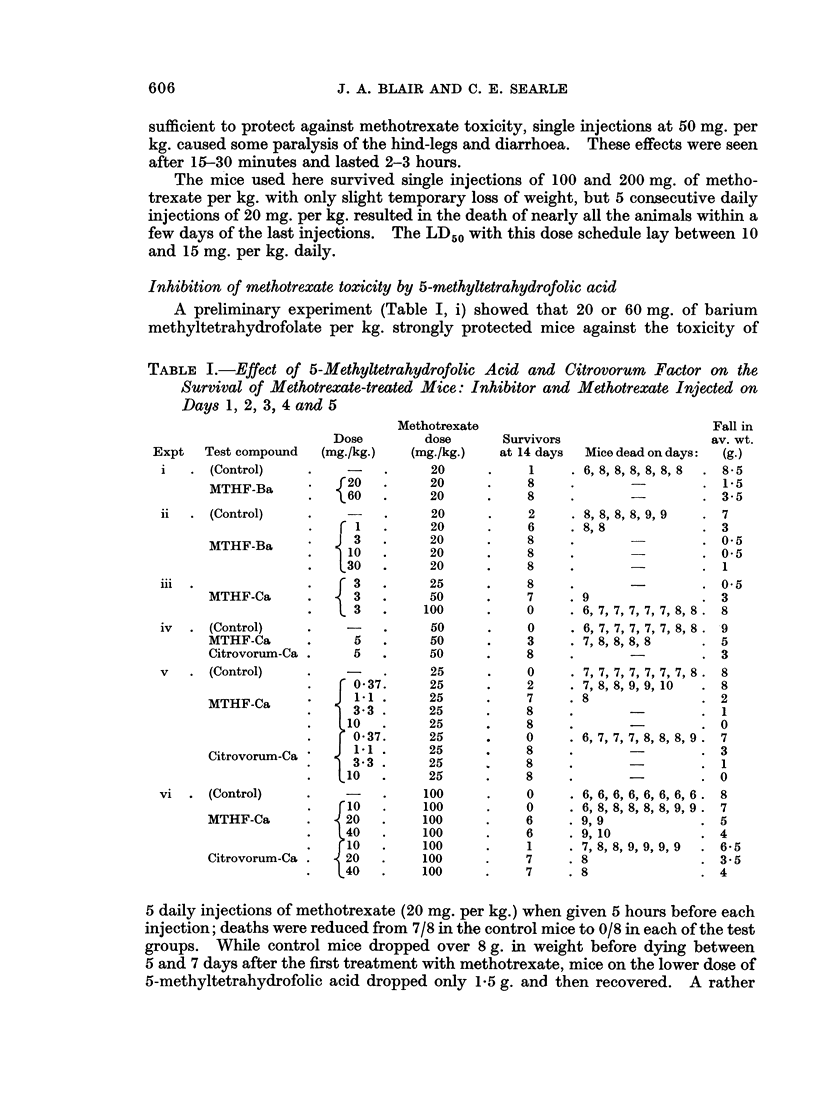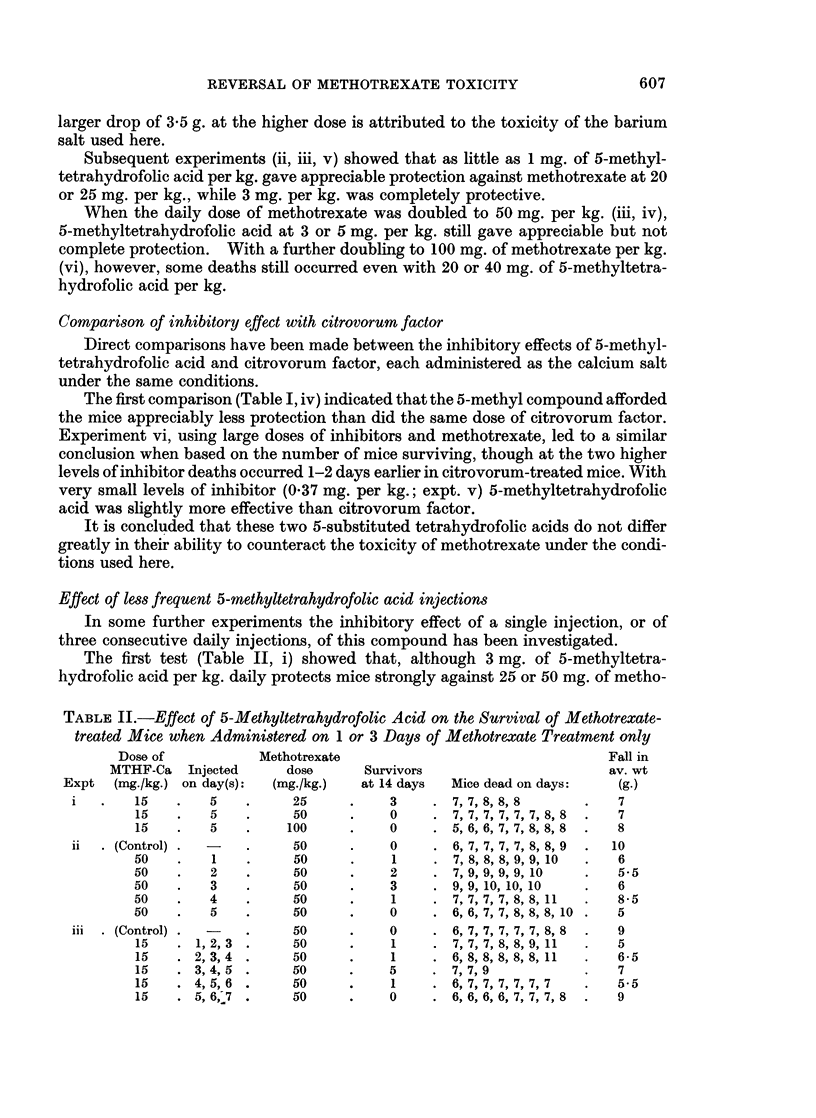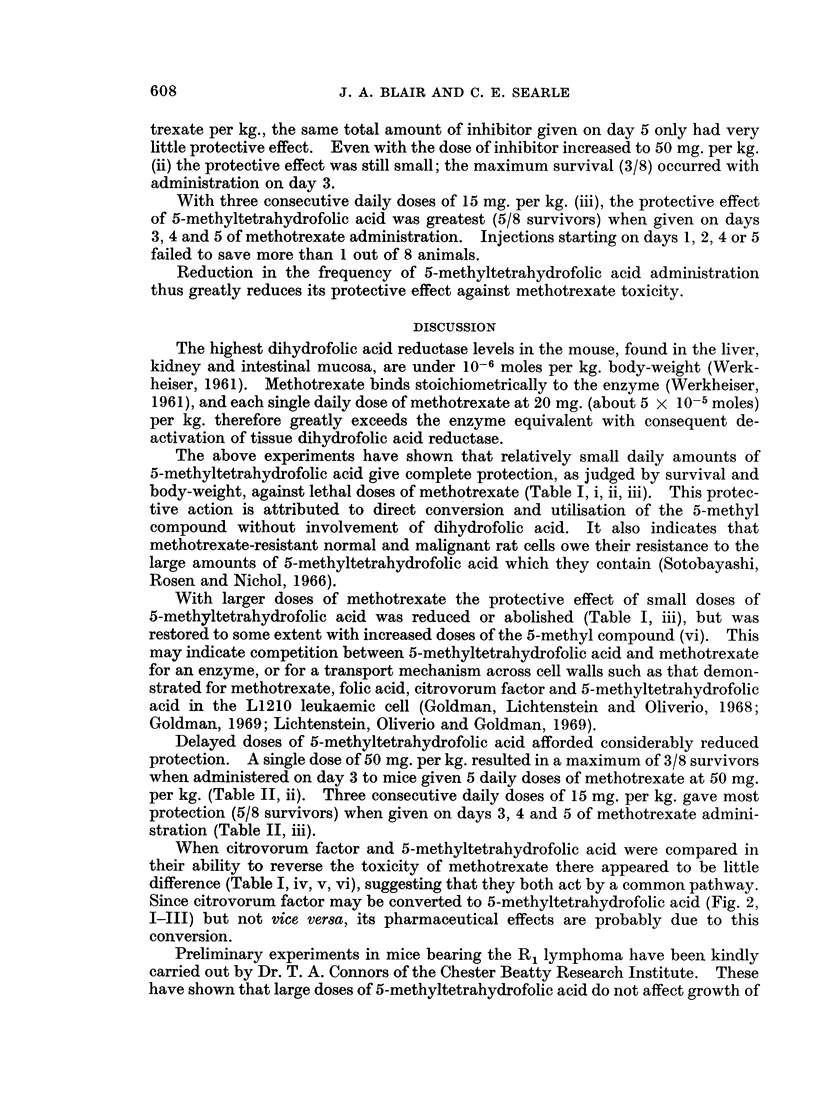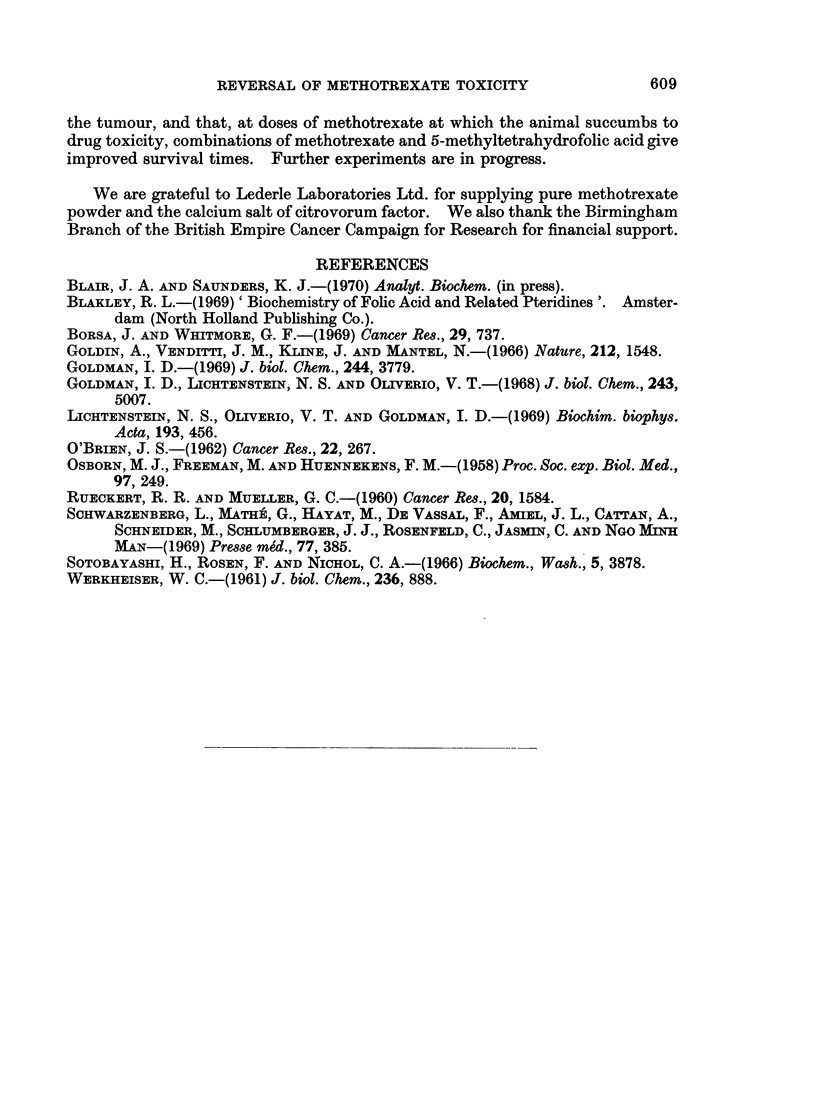# Reversal of Methotrexate Toxicity in Mice by 5-Methyltetrahydrofolic Acid

**DOI:** 10.1038/bjc.1970.73

**Published:** 1970-09

**Authors:** J. A. Blair, C. E. Searle

## Abstract

1. Mice have been compeletly protected against the lethal effects of repeated injections of methotrexate by 5-methyltetrahydrofolic acid. Methotrexate-resistant cells probably owe their resistance to their high content of this substance.

2. Daily small doses were much more effective than fewer but larger doses.

3. The reduced efficiency found in counteracting high dosages of methotrexate may be due to interference with folate transport across cell walls.

4. 5-Methyltetrahydrofolic acid and citrovorum factor did not differ greatly in their ability to inhibit methotre toxicity. It is suggested that the pharmaceutical effects of citrovorum factor are due to its conversion to 5-methyltetrahydrofolic acid.


### REVERSAL OF METHOTREXATE TOXICITY IN MICE BY 5-METHYLTETRAHYDROFOLIC ACID

J. A. BLAIR AND C. E. SEARLE

*From the Department of Chemistry, University of Aston in Birmingham, Birmingham* 4, *and the Department of Cancer Studies, University of Birmingham, Birmingham* 15

Received for publication May 26, 1970

**SUMMARY.—1. Mice have been completely protected against the lethal effects of repeated injections of methotrexate by 5-methyltetrahydrofolic acid. Methotrexate-resistant cells probably owe their resistance to their high content of this substance.**

**2. Daily small doses were much more effective than fewer but larger doses.**

**3. The reduced efficiency found in counteracting high dosages of methotrexate may be due to interference with folate transport across cell walls.**

**4. 5-Methyltetrahydrofolic acid and citrovorum factor did not differ greatly in their ability to inhibit methotrexate toxicity. It is suggested that the pharmaceutical effects of citrovorum factor are due to its conversion to 5-methyltetrahydrofolic acid.**

ALTHOUGH methotrexate is a powerful inhibitor of malignant cell growth its clinical use is restricted by its toxicity, the dose required for complete destruction of the malignant cells usually being sufficient to destroy the host. Attempts have been made to improve its clinical effectiveness by using agents and modes of treatment which reduce the overall lethal effects while retaining the tumour inhibitory properties. Of these, citrovorum factor (folinic acid; 5-formyltetrahydrofolic acid; DL-5-formyl-5,6,7,8-tetrahydropteroyl-L-monoglutamic acid) has proved the most useful. Combinations of large doses of methotrexate and citrovorum factor give improved survival rates in leukaemic mice (Goldin, Venditti, Kline and Mantel, 1966) and appear advantageous in the treatment of human tumours (Schwarzenberg *et al.*, 1969). Citrovorum factor is also used as an antidote for methotrexate poisoning. The main storage form of folates in the body being 5-methyltetrahydrofolic acid (Blakley, 1969), it was anticipated that this compound would have similar protective properties.

The cytotoxic effects of methotrexate have been previously studied by Rueckert and Mueller (1960) and O'Brien (1962). The conversion of deoxyuridylate to thymidylate (for incorporation into DNA) utilises methylenetetrahydrofolate, and the repletion of this pool of folate requires the production of tetrahydrofolic acid by dihydrofolic acid reductase (Fig. 1). Methotrexate irreversibly inhibits the reduction of dihydrofolic acid and thus depletes the cell store of tetrahydrofolic acid. This inhibits synthesis of DNA since methylation of deoxyuridylate to thymidylate no longer occurs (Osborn, Freeman and Huennekens, 1958; O'Brien, 1962). Since tetrahydrofolic acid is also converted (Fig. 2) to 10-formyltetrahydrofolic acid which is required for purine biosynthesis, the cellular synthesis of these compounds is also reduced (Blakley, 1969; Borsa and Whitmore, 1969).

FIG. 1.—Utilisation of folates in DNA synthesis.

FIG. 2.—Utilisation of citrovorum factor in the cell folate cycle.

The citrovorum factor can enter the folate metabolic cycle directly without prior reduction by dihydrofolic acid reductase. Several routes are possible, but no evidence is yet available to decide which are used. In all routes citrovorum factor is converted to the 5,10-methenyl compound (Fig. 2, I). This is reduced (II) to 5,10-methylenetetrahydrofolic acid which is then available for the methylation of deoxyuridylate (Fig. 1). The 5,10-methylene compound may be further reduced (Fig. 2, III) to 5-methyltetrahydrofolic acid, which can be converted (IV) to tetrahydrofolic acid by vitamin $B_{12}$ demethylation. 5,10-Methenyltetrahydrofolic acid is also hydrolysed (V) to 10-formyltetrahydrofolic acid, which loses the formyl group in purine biosynthesis (VI) forming tetrahydrofolic acid (Blakley, 1969).

Irrespective of the route followed, citrovorum factor maintains the cell store of tetrahydrofolates without utilisation of the dihydrofolic acid reductase pathway, and thus allows synthesis of DNA and purines in a cell in which the normal route is blocked by methotrexate. The effect of the citrovorum factor will be the same whether it is administered before or after methotrexate. This bypassing of dihydrofolic acid reductase probably accounts for the reversal of the toxic effects of methotrexate by the citrovorum factor.

A convenient synthesis of DL-5-methyltetrahydrofolic acid in good yield now being available (Blair and Saunders, 1970), its effect on methotrexate toxicity in mice has been studied. Since 5-methyltetrahydrofolic acid replenishes the tetrahydrofolate store within the cell by only one route (Fig. 2, IV), the relative efficiencies of citrovorum factor and 5-methyltetrahydrofolic acid have also been compared to elucidate the mode of action of the former.

### EXPERIMENTAL

### *Compounds*

*Methotrexate.*—For experiments i and ii (Table I) methotrexate sodium parenteral (Lederle Laboratories Division) was used after dilution to the appropriate concentrations of methotrexate and NaCl. Subsequently, pure methotrexate dihydrate (Lederle) was added to 0·9 per cent NaCl and dissolved by adding the minimum amount of solid $NaHCO_3$ (final pH approximately 8). Small amounts of residual gelatinous material noticed in some samples were removed by filtration or centrifugation.

DL-5-*Methyl*-5,6,7,8-*tetrahydrofolic acid.*—This was synthetic material (Blair and Saunders, 1970) which for the first tests was only available as the barium salt (MTHF-Ba in tables). After this the calcium salt (MTHF-Ca) was employed instead. Both were administered within an hour of dissolution in 0·9 per cent NaCl.

*Citrovorum factor.*—This was used as a fresh solution of the calcium salt (calcium leucovorin, Lederle) in 0·9 per cent NaCl.

All test solutions were administered subcutaneously with the dose per kg. bodyweight dissolved in 10 ml., *i.e.* the volume injected was 0·3 ml. per 30 g. mouse. Control mice received the same volume of saline solution.

### *Animals*

Male C57BL/Bcr × IF/Bcr $F_1$ hybrid mice were used throughout. They were housed in plastic (" Perspex ") boxes each containing 4 mice, and were fed cube diet 41B and tap water *ad libitum.*

Groups comprised 8 mice, each weighing 27–29 g. at the time of the first injections. They were weighed several times before starting the experiments, and daily during the experiments. Deaths were also recorded daily. All mice which survived beyond day 11 recovered, and were killed 2–3 weeks after the last injections.

In all experiments methotrexate was administered on 5 consecutive afternoons (days 1–5), and the test compounds were injected 5 hours earlier into the opposite flanks of the animals. The dosages and results obtained are summarised in Table I. In some experiments (Table II) the test compounds were administered on one day or on three consecutive days only.

### RESULTS

### *Toxicity of compounds*

Calcium 5-methyltetrahydrofolate and citrovorum factor showed no toxic effects at any dose used. Though the barium salt was also not toxic at doses sufficient to protect against methotrexate toxicity, single injections at 50 mg. per kg. caused some paralysis of the hind-legs and diarrhoea. These effects were seen after 15–30 minutes and lasted 2–3 hours.

The mice used here survived single injections of 100 and 200 mg. of methotrexate per kg. with only slight temporary loss of weight, but 5 consecutive daily injections of 20 mg. per kg. resulted in the death of nearly all the animals within a few days of the last injections. The $LD_{50}$ with this dose schedule lay between 10 and 15 mg. per kg. daily.

*Inhibition of methotrexate toxicity by 5-methyltetrahydrofolic acid*

A preliminary experiment (Table I, i) showed that 20 or 60 mg. of barium methyltetrahydrofolate per kg. strongly protected mice against the toxicity of

TABLE I.—*Effect of 5-Methyltetrahydrofolic Acid and Citrovorum Factor on the Survival of Methotrexate-treated Mice: Inhibitor and Methotrexate Injected on Days* 1, 2, 3, 4 *and* 5

| Expt | Test compound | Dose (mg./kg.) | Methotrexate dose (mg./kg.) | Survivors at 14 days | Mice dead on days: | Fall in av. wt. (g.) |
|---|---|---|---|---|---|---|
| i | (Control) | — | 20 | 1 | 6, 8, 8, 8, 8, 8, 8 | 8·5 |
| | MTHF-Ba | 20 | 20 | 8 | — | 1·5 |
| | | 60 | 20 | 8 | — | 3·5 |
| ii | (Control) | — | 20 | 2 | 8, 8, 8, 8, 9, 9 | 7 |
| | MTHF-Ba | 1 | 20 | 6 | 8, 8 | 3 |
| | | 3 | 20 | 8 | — | 0·5 |
| | | 10 | 20 | 8 | — | 0·5 |
| | | 30 | 20 | 8 | — | 1 |
| iii | MTHF-Ca | 3 | 25 | 8 | — | 0·5 |
| | | 3 | 50 | 7 | 9 | 3 |
| | | 3 | 100 | 0 | 6, 7, 7, 7, 7, 7, 8, 8 | 8 |
| iv | (Control) | — | 50 | 0 | 6, 7, 7, 7, 7, 7, 8, 8 | 9 |
| | MTHF-Ca | 5 | 50 | 3 | 7, 8, 8, 8, 8 | 5 |
| | Citrovorum-Ca | 5 | 50 | 8 | — | 3 |
| v | (Control) | — | 25 | 0 | 7, 7, 7, 7, 7, 7, 7, 8 | 8 |
| | MTHF-Ca | 0·37 | 25 | 2 | 7, 8, 8, 9, 9, 10 | 8 |
| | | 1·1 | 25 | 7 | 8 | 2 |
| | | 3·3 | 25 | 8 | — | 1 |
| | | 10 | 25 | 8 | — | 0 |
| | Citrovorum-Ca | 0·37 | 25 | 0 | 6, 7, 7, 7, 8, 8, 8, 9 | 7 |
| | | 1·1 | 25 | 8 | — | 3 |
| | | 3·3 | 25 | 8 | — | 1 |
| | | 10 | 25 | 8 | — | 0 |
| vi | (Control) | — | 100 | 0 | 6, 6, 6, 6, 6, 6, 6, 6 | 8 |
| | MTHF-Ca | 10 | 100 | 0 | 6, 8, 8, 8, 8, 8, 9, 9 | 7 |
| | | 20 | 100 | 6 | 9, 9 | 5 |
| | | 40 | 100 | 6 | 9, 10 | 4 |
| | Citrovorum-Ca | 10 | 100 | 1 | 7, 8, 8, 9, 9, 9, 9 | 6·5 |
| | | 20 | 100 | 7 | 8 | 3·5 |
| | | 40 | 100 | 7 | 8 | 4 |

5 daily injections of methotrexate (20 mg. per kg.) when given 5 hours before each injection; deaths were reduced from 7/8 in the control mice to 0/8 in each of the test groups. While control mice dropped over 8 g. in weight before dying between 5 and 7 days after the first treatment with methotrexate, mice on the lower dose of 5-methyltetrahydrofolic acid dropped only 1·5 g. and then recovered. A rather larger drop of 3·5 g. at the higher dose is attributed to the toxicity of the barium salt used here.

Subsequent experiments (ii, iii, v) showed that as little as 1 mg. of 5-methyltetrahydrofolic acid per kg. gave appreciable protection against methotrexate at 20 or 25 mg. per kg., while 3 mg. per kg. was completely protective.

When the daily dose of methotrexate was doubled to 50 mg. per kg. (iii, iv), 5-methyltetrahydrofolic acid at 3 or 5 mg. per kg. still gave appreciable but not complete protection. With a further doubling to 100 mg. of methotrexate per kg. (vi), however, some deaths still occurred even with 20 or 40 mg. of 5-methyltetrahydrofolic acid per kg.

*Comparison of inhibitory effect with citrovorum factor*

Direct comparisons have been made between the inhibitory effects of 5-methyltetrahydrofolic acid and citrovorum factor, each administered as the calcium salt under the same conditions.

The first comparison (Table I, iv) indicated that the 5-methyl compound afforded the mice appreciably less protection than did the same dose of citrovorum factor. Experiment vi, using large doses of inhibitors and methotrexate, led to a similar conclusion when based on the number of mice surviving, though at the two higher levels of inhibitor deaths occurred 1–2 days earlier in citrovorum-treated mice. With very small levels of inhibitor (0·37 mg. per kg.; expt. v) 5-methyltetrahydrofolic acid was slightly more effective than citrovorum factor.

It is concluded that these two 5-substituted tetrahydrofolic acids do not differ greatly in their ability to counteract the toxicity of methotrexate under the conditions used here.

*Effect of less frequent 5-methyltetrahydrofolic acid injections*

In some further experiments the inhibitory effect of a single injection, or of three consecutive daily injections, of this compound has been investigated.

The first test (Table II, i) showed that, although 3 mg. of 5-methyltetrahydrofolic acid per kg. daily protects mice strongly against 25 or 50 mg. of metho-

TABLE II.—*Effect of 5-Methyltetrahydrofolic Acid on the Survival of Methotrexate-treated Mice when Administered on 1 or 3 Days of Methotrexate Treatment only*

| Expt | Dose of MTHF-Ca (mg./kg.) | Injected on day(s): | Methotrexate dose (mg./kg.) | Survivors at 14 days | Mice dead on days: | Fall in av. wt (g.) |
|---|---|---|---|---|---|---|
| i | 15 | 5 | 25 | 3 | 7, 7, 8, 8, 8 | 7 |
| | 15 | 5 | 50 | 0 | 7, 7, 7, 7, 7, 7, 8, 8 | 7 |
| | 15 | 5 | 100 | 0 | 5, 6, 6, 7, 7, 8, 8, 8 | 8 |
| ii | (Control) | — | 50 | 0 | 6, 7, 7, 7, 7, 8, 8, 9 | 10 |
| | 50 | 1 | 50 | 1 | 7, 8, 8, 8, 9, 9, 10 | 6 |
| | 50 | 2 | 50 | 2 | 7, 9, 9, 9, 9, 10 | 5·5 |
| | 50 | 3 | 50 | 3 | 9, 9, 10, 10, 10 | 6 |
| | 50 | 4 | 50 | 1 | 7, 7, 7, 7, 8, 8, 11 | 8·5 |
| | 50 | 5 | 50 | 0 | 6, 6, 7, 7, 8, 8, 8, 10 | 5 |
| iii | (Control) | — | 50 | 0 | 6, 7, 7, 7, 7, 7, 8, 8 | 9 |
| | 15 | 1, 2, 3 | 50 | 1 | 7, 7, 7, 8, 8, 9, 11 | 5 |
| | 15 | 2, 3, 4 | 50 | 1 | 6, 8, 8, 8, 8, 8, 11 | 6·5 |
| | 15 | 3, 4, 5 | 50 | 5 | 7, 7, 9 | 7 |
| | 15 | 4, 5, 6 | 50 | 1 | 6, 7, 7, 7, 7, 7, 7 | 5·5 |
| | 15 | 5, 6, 7 | 50 | 0 | 6, 6, 6, 6, 7, 7, 7, 8 | 9 |

trexate per kg., the same total amount of inhibitor given on day 5 only had very little protective effect. Even with the dose of inhibitor increased to 50 mg. per kg. (ii) the protective effect was still small; the maximum survival (3/8) occurred with administration on day 3.

With three consecutive daily doses of 15 mg. per kg. (iii), the protective effect of 5-methyltetrahydrofolic acid was greatest (5/8 survivors) when given on days 3, 4 and 5 of methotrexate administration. Injections starting on days 1, 2, 4 or 5 failed to save more than 1 out of 8 animals.

Reduction in the frequency of 5-methyltetrahydrofolic acid administration thus greatly reduces its protective effect against methotrexate toxicity.

### DISCUSSION

The highest dihydrofolic acid reductase levels in the mouse, found in the liver, kidney and intestinal mucosa, are under $10^{-6}$ moles per kg. body-weight (Werkheiser, 1961). Methotrexate binds stoichiometrically to the enzyme (Werkheiser, 1961), and each single daily dose of methotrexate at 20 mg. (about $5 \times 10^{-5}$ moles) per kg. therefore greatly exceeds the enzyme equivalent with consequent deactivation of tissue dihydrofolic acid reductase.

The above experiments have shown that relatively small daily amounts of 5-methyltetrahydrofolic acid give complete protection, as judged by survival and body-weight, against lethal doses of methotrexate (Table I, i, ii, iii). This protective action is attributed to direct conversion and utilisation of the 5-methyl compound without involvement of dihydrofolic acid. It also indicates that methotrexate-resistant normal and malignant rat cells owe their resistance to the large amounts of 5-methyltetrahydrofolic acid which they contain (Sotobayashi, Rosen and Nichol, 1966).

With larger doses of methotrexate the protective effect of small doses of 5-methyltetrahydrofolic acid was reduced or abolished (Table I, iii), but was restored to some extent with increased doses of the 5-methyl compound (vi). This may indicate competition between 5-methyltetrahydrofolic acid and methotrexate for an enzyme, or for a transport mechanism across cell walls such as that demonstrated for methotrexate, folic acid, citrovorum factor and 5-methyltetrahydrofolic acid in the L1210 leukaemic cell (Goldman, Lichtenstein and Oliverio, 1968; Goldman, 1969; Lichtenstein, Oliverio and Goldman, 1969).

Delayed doses of 5-methyltetrahydrofolic acid afforded considerably reduced protection. A single dose of 50 mg. per kg. resulted in a maximum of 3/8 survivors when administered on day 3 to mice given 5 daily doses of methotrexate at 50 mg. per kg. (Table II, ii). Three consecutive daily doses of 15 mg. per kg. gave most protection (5/8 survivors) when given on days 3, 4 and 5 of methotrexate administration (Table II, iii).

When citrovorum factor and 5-methyltetrahydrofolic acid were compared in their ability to reverse the toxicity of methotrexate there appeared to be little difference (Table I, iv, v, vi), suggesting that they both act by a common pathway. Since citrovorum factor may be converted to 5-methyltetrahydrofolic acid (Fig. 2, I–III) but not *vice versa*, its pharmaceutical effects are probably due to this conversion.

Preliminary experiments in mice bearing the $R_1$ lymphoma have been kindly carried out by Dr. T. A. Connors of the Chester Beatty Research Institute. These have shown that large doses of 5-methyltetrahydrofolic acid do not affect growth of the tumour, and that, at doses of methotrexate at which the animal succumbs to drug toxicity, combinations of methotrexate and 5-methyltetrahydrofolic acid give improved survival times. Further experiments are in progress.

We are grateful to Lederle Laboratories Ltd. for supplying pure methotrexate powder and the calcium salt of citrovorum factor. We also thank the Birmingham Branch of the British Empire Cancer Campaign for Research for financial support.

### REFERENCES

BLAIR, J. A. AND SAUNDERS, K. J.—(1970) *Analyt. Biochem.* (in press).
BLAKLEY, R. L.—(1969) 'Biochemistry of Folic Acid and Related Pteridines'. Amsterdam (North Holland Publishing Co.).
BORSA, J. AND WHITMORE, G. F.—(1969) *Cancer Res.*, **29**, 737.
GOLDIN, A., VENDITTI, J. M., KLINE, J. AND MANTEL, N.—(1966) *Nature*, **212**, 1548.
GOLDMAN, I. D.—(1969) *J. biol. Chem.*, **244**, 3779.
GOLDMAN, I. D., LICHTENSTEIN, N. S. AND OLIVERIO, V. T.—(1968) *J. biol. Chem.*, **243**, 5007.
LICHTENSTEIN, N. S., OLIVERIO, V. T. AND GOLDMAN, I. D.—(1969) *Biochim. biophys. Acta*, **193**, 456.
O'BRIEN, J. S.—(1962) *Cancer Res.*, **22**, 267.
OSBORN, M. J., FREEMAN, M. AND HUENNEKENS, F. M.—(1958) *Proc. Soc. exp. Biol. Med.*, **97**, 249.
RUECKERT, R. R. AND MUELLER, G. C.—(1960) *Cancer Res.*, **20**, 1584.
SCHWARZENBERG, L., MATHÉ, G., HAYAT, M., DE VASSAL, F., AMIEL, J. L., CATTAN, A., SCHNEIDER, M., SCHLUMBERGER, J. J., ROSENFELD, C., JASMIN, C. AND NGO MINH MAN—(1969) *Presse méd.*, **77**, 385.
SOTOBAYASHI, H., ROSEN, F. AND NICHOL, C. A.—(1966) *Biochem., Wash.*, **5**, 3878.
WERKHEISER, W. C.—(1961) *J. biol. Chem.*, **236**, 888.